# Loss of PPM1A expression enhances invasion and the epithelial-to-mesenchymal transition in bladder cancer by activating the TGF-β/Smad signaling pathway

**DOI:** 10.18632/oncotarget.2144

**Published:** 2014-07-01

**Authors:** Jiang Geng, Jie Fan, Qi Ouyang, Xiaopeng Zhang, Xiaolong Zhang, Juan Yu, Zude Xu, Qianyu Li, Xudong Yao, Xiuping Liu, Junhua Zheng

**Affiliations:** ^1^ Department of Urology, Tenth People's Hospital; Tongji University, Shanghai, China; ^2^ Department of Pathology, School of Basic Medical Sciences, Fudan University, Shanghai, China; ^3^ Department of Pathology, Huashan Hospital; Fudan University, Shanghai, China; ^4^ Department of Pathology, Fifth People's Hospital, Fudan University, Shanghai, China; ^5^ Department of Pathology, Tenth People's Hospital; Tongji University, Shanghai, China

**Keywords:** urinary bladder cancer, PPM1A, TGF-β signaling, muscle-invasive, EMT

## Abstract

The transforming growth factor-β (TGF-β) signaling pathway is believed to contribute to carcinoma development by increasing cell invasiveness and metastasis and inducing the epithelial-to-mesenchymal transition (EMT). Protein phosphatase PPM1A has been reported to dephosphorylate TGF-β-activated Smad2/3, thus inhibiting the TGF-β signaling pathway. In this study, we investigated the role of PPM1A in bladder cancer. PPM1A protein expression was analyzed in 145 bladder cancer specimens. The loss of PPM1A expression was predictive of poor survival and high muscle-invasiveness. PPM1A was more commonly deficient among muscle-invasive relapse samples compared to primary tumors in twenty paired bladder cancer tissues. Functional studies indicated that blockade of PPM1A through lentivirus-mediated RNA interference significantly promoted urinary bladder cancer (BCa) cell motility, the EMT *in vitro* and metastasis *in vivo*, and these effects were dependent on the TGF-β/Smad signaling pathway. The increase in p-Smad2/3 induced by TGF-β1 correlated with the degree of PPM1A depletion in BCa cells, which resulted in an altered expression profile of TGF-β-inducible genes. The correlations between PPM1A and biomarkers related to the TGF-β signaling pathway and tumor invasion were also detected in BCa samples. These results demonstrate that loss of PPM1A is associated with the development of tumor invasion in bladder cancer.

## INTRODUCTION

Urinary bladder cancer (BCa) is the most common malignancy of the urinary tract, with 73,000 new cases and 15,210 deaths in 2013 in the US alone [1]. Urothelial carcinoma represents approximately 90% of BCas that arise from an epithelial origin. BCa is classified into superficial (pTa, pT1 and CIS) and muscle-invasive (pT2-4) cancer based on whether the tumor infiltration extends to the muscular bladder wall [2]. Non-invasive BCa has a high risk of recurrence, and 1/3 of patients will progress to muscle invasion [3, 4]. Muscle-invasive BCa is clinically unfavorable, with a 5-year overall survival rate of 48% to 67% [5]. To date, the molecular mechanism underlying muscle-invasive BCa remains unknown.

Tumor invasiveness and metastasis involve biological cascades consisting of multiple steps, including the loss of cellular adhesion, increased motility and invasiveness, entry and survival in the circulation, exit into new tissue and the eventual colonization at a distant site. The TGF-β signaling pathway is believed to contribute to carcinoma development by increasing cancer cell motility, invasiveness and metastasis and inducing the epithelial-to-mesenchymal transition (EMT) [6, 7]. However, this signaling pathway has a dual effect on tumor growth, with both tumor-suppressing and tumor-promoting activities, depending on the stage of carcinogenesis and cell type involved. Previous studies have also shown that the TGF-β pathway plays important roles in the tumorigenesis of BCa [8], and plasma TGF-β1 levels were markedly elevated in patients with muscle-invasive BCa; the highest plasma TGF-β1 levels were observed in patients with bladder carcinoma that had metastasized to the lymph nodes[9]. The TGF-β-induced Smad signaling pathway has been studied extensively in an effort to understand the complex and versatile responses governing tumor metastasis, increased motility, invasiveness and the EMT [10-12]. As the TGF-β signaling pathway influences such diverse biological events, it is logical and definite that tight regulations exist to control TGF-β signaling pathway activation.

PPM1A (protein phosphatase, Mg^2+^/Mn^2+^ dependent 1A) is a member of the PP2C family of Ser/Thr protein phosphatases [13], and it has been shown to dephosphorylate TGF-β-activated Smad2/3, thus enhancing disassembly of the activated Smad complex and promoting the nuclear export of dephosphorylated Smad2/3 [14, 15]. PPM1A has also been implicated in the regulation of cell invasion and migration [16]. Moreover, further studies also imply that PPM1A is an important tumor suppression factor [17, 18]. As the precise role of PPM1A in BCa remains largely unknown, we hypothesized that PPM1A may be regulating the TGF-β/Smad signaling pathway and involved in BCa development and progression. In this study, we investigated the clinicopathologic significance and potential role of PPM1A in invasion and the EMT in BCa.

## RESULTS

### Decreased PPM1A expression was associated with muscle invasion and poor prognosis of BCa

**Figure 1:**
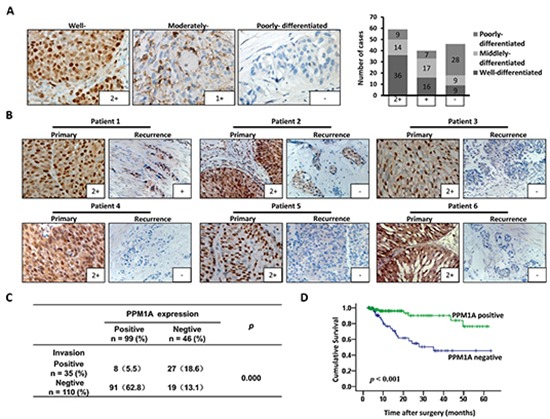
PPM1A expression correlated with prognosis and muscle invasion in patients with BCa **(A)** Representative IHC staining of the PPM1A protein expression in bladder cancer tissues of different histological differentiation grades is shown (× 400). **(B)** Matched pairs of primary tumors and recurrent BCa samples were analyzed for PPM1A protein expression. The expression of PPM1A in recurrent muscle-invasive BCa was significantly lower than that of primary non-muscle-invasive BCa (× 200). **(C)** Correlation between the expression of PPM1A and tumor muscle invasion in 145 BCa samples. **(D)** Kaplan-Meier curves with log-rank analyses for BCa patients with negative PPM1A-expressing tumors (n = 46) versus positive PPM1A-expressing tumors (n = 99).

We first assessed PPM1A expression using an IHC assay in 145 cases of BCa. Positive immunostaining for PPM1A was observed in the nuclei of BCa cells, with or without cytoplasmic staining. Tumors were considered negative for PPM1A expression when there was no nuclear staining or staining in < 5% of the tumor cells (n = 46), positive staining in ≥ 5% of the neoplastic cells was considered nuclear staining (n = 99). PPM1A expression was lost in 18.8% (17/90) of the superficial BCa and in 52.7% of the muscle-invasive cancer samples. Fig1A shows that the expression of PPM1A was higher in well differentiated tumors, lower in moderately differentiated tissues, and lost in most of the poorly differentiated tumors. Correlation analyses indicated that histological grade was significantly associated with the loss of PPM1A expression (*p* = 0.012) (Supplementary Table 1). These results indicate that in BCa, the loss of PPM1A is significantly associated with poorly differentiated tumors.

Furthermore, 20 matched pairs of primary tumors and recurrent bladder cancer samples were analyzed for PPM1A protein expression (Figure 1B). The information of these patients was shown in Supplementary Table 2. The PPM1A protein expression differed between primary superficial tumors and muscle-invasive recurrent cancer tissues with more negative staining among the recurrence samples (*p* = 0.012). We found that PPM1A expression was significantly lower in muscle-invasive recurrence compared to primary carcinomas *in situ* (Figure 1C). In recurrent non-muscle-invasive BCa tissues and paired primary tumors, there was no significant difference in the expression of PPM1A (Supplementary Table 2). Consistent with these findings, we observed that there was a significant correlation between PPM1A expression and muscle invasion in the 145-patient cohort (*p* < 0.01). Muscle-invasive cancer presented significantly lower levels of PPM1A than non-invasive BCa (pTa, pT1 and CIS) (*p* < 0.01) (Figure 1D).

Additionally, as shown in Figure 1E, Kaplan–Meier survival curves and the log-rank test Survival analysis showed that the overall survival of patients with negative PPM1A expression was significantly poorer than that of patients with a high level of PPM1A (*p* < 0.001) (Fig. 1E). PPM1A expression was strongly associated with the tumor stage (*p* = 0.038) (Supplementary Table 1). These results suggested that the loss of PPM1A expression may be a poor prognostic factor for survival in BCa patients and may mediate more aggressive characteristics, such as poor differentiation and high muscle invasiveness.

### Downregulation of PPM1A significantly promoted the invasive capacity of BCa cells *in vitro* in a TGF-β1-dependent manner

**Figure 2:**
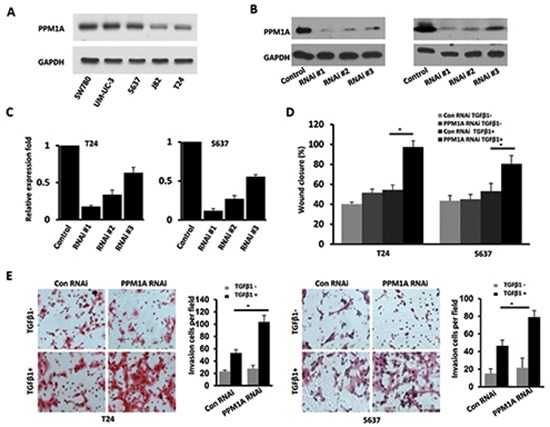
Downregulation of PPM1A significantly promoted cellular invasion, which was dependent on TGF-β1, *in vitro* **(A)** Western blotting analysis of PPM1A expression in five bladder cancer cell lines. Establishment and selection of notably and stably PPM1A-silenced T24 and 5637 cells, Western blotting. **(B)** and real-time RT-PCR. **(C)** were performed to detect the protein and mRNA expression. **(D)** The effect of PPM1A knockdown on the migration rates of BCa cells treated with TGF-β1 (200 pM) or vehicle was compared in wound healing assays in T24 and 5637 cells. **(E)** The invasive capacity of BCa cells treated with TGF-β1 (200 pM) or vehicle was compared using Matrigel invasion assays in T24 and 5637 cells.

Our clinical findings suggested that PPM1A could be an important molecule that regulates muscle invasion in BCa. As such, we sought to further study the role of PPM1A in BCa cells. We assessed PPM1A expression and the levels of activation of Smad2 and Smad3 using Western blotting in a panel of BCa cell lines (Figure 2A and Supplementary Figure 1A). We found that PPM1A was expressed in all 5 BCa cell lines, and 5637 and T24 cells were chosen for further PPM1A studies. Three RNAi sequences targeting human PPM1A were used to generate cells with stable knockdown of PPM1A expression. Western blot and RT-PCR analysis revealed that one of the lentiviral small interfering RNAs (RNAi #1) was able to reduce endogenous PPM1A protein expression by over 80% relative to the level in control cells (Figure 2B and Figure 2C).

To assess our hypothesis that PPM1A regulates BCa invasiveness, we evaluated the effect of PPM1A knockdown on BCa cell migration and invasion using a wound closure assay and transwell chambers with Matrigel coating. The wound closure assay demonstrated that PPM1A knockdown cells showed a significantly faster rate of wound closure compared to control RNAi cells only when TGF-β1 was present; in the absence of TGF-β1 treatment, there was no significant change compared to the control cells (Figure 2D). Consistent with these results, the transwell chamber assay also indicated that with TGF-β1 treatment, PPM1A knockdown significantly increased BCa cell invasion (Figure 2E). Taken together, these data indicate that in the presence of TGF-β1, decreased PPM1A expression significantly promoted BCa cell migration/invasion *in vitro*.

### Downregulation of PPM1A significantly promoted cellular growth, invasion and metastasis *in vivo*

**Figure 3:**
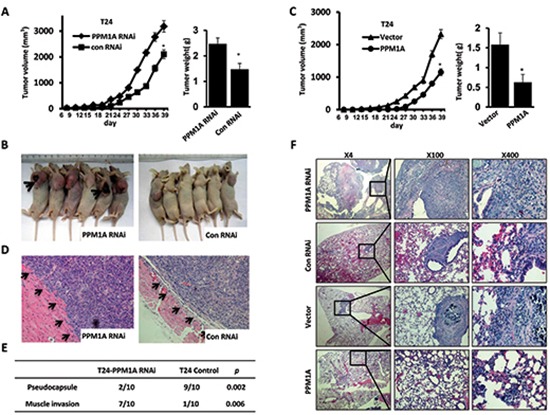
PPM1A suppressed tumor cell invasion and metastasis in BCa cells *in vivo* (**A** and **B**) The effect of PPM1A on tumor growth was evaluated in a nude mouse xenograft model, T24 control and T24-PPM1A-RNAi (loss expression of PPM1A) cells were xenografted and the weights of tumors formed in the xenograft model. **(C)** The effect of PPM1A on tumor growth was evaluated in a nude mouse xenograft model, T24 vector, T24-PPM1A (high expression of PPM1A) cells were xenografted, and the weights of tumors formed in the xenograft model. (**D** and **E**) At postmortem examination, the pseudo-capsules of the xenograft tumors formed from the T24-PPM1A-RNAi (low-expression PPM1A cell) cells were absent, whereas the xenograft tumors formed from T24 control PPM1A-RNAi cells demonstrated a smooth surface with well-formed pseudo-capsules. **(F)** Lung metastasis assay *in vivo*. The metastatic lesions in the mice's lungs were captured in T24-PPM1A-RNAi, T24-PPM1A and control T24 cell lines.

To investigate the effect of PPM1A expression on BCa cell growth, invasion and metastasis *in vivo*, we xenografted the following cell types into nude mice: T24 vector, T24 PPM1A (high expression of PPM1A), T24 control and T24 PPM1A RNAi (low expression of PPM1A). At a postmortem examination conducted after 39 days, we found that tumors derived from T24 PPM1A RNAi cells grew much faster and weighed significantly more than those formed from T24 control cells and PPM1A overexpressing cells (Figure 3A, Figure 3B). Meanwhile, overexpression of PPM1A significantly suppressed tumor growth relative to the growth of T24 PPM1A RNAi cells and vector control cells (Figure 3C). Furthermore, in xenograft tumors formed from T24 PPM1A RNAi cells, the tumor margin was not continuous and was even absent in most cases; as a result, these tumors were more likely to infiltrate into the surrounding tissue (*p* < 0.05) (Figure 3D, Figure 3E).

To further study the role of PPM1A in BCa cell metastasis *in vivo*, we injected T24 vector, T24 PPM1A, T24 RNAi control and T24 PPM1A RNAi cells into the tail veins of nude mice and compared the resulting metastatic nodules that formed in the lungs. Seven weeks after injection, mice injected with PPM1A-knockdown cells developed significantly more lung metastases than mice injected with T24 PPM1A and control cells (Figure 3F, Supplementary Figure 1B). Collectively, our results indicate that the loss of PPM1A can promote the tumorigenicity, invasiveness and metastatic potential of BCa cells *in vivo*.

### PPM1A reduced TGF-β signaling in BCa cells by dephosphorylating TGF-β-activated Smad2/3

**Figure 4:**
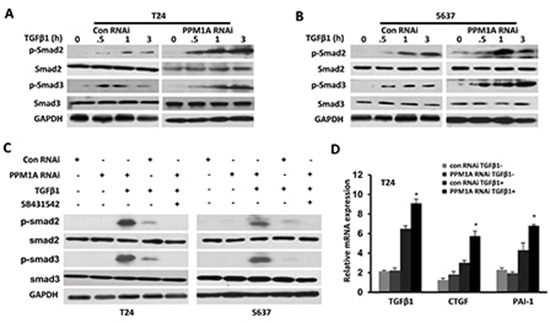
PPM1A terminated TGF-β signaling in BCa cells by dephosphorylating TGF-β-activated Smad2/3 PPM1A was silenced in T24 and 5637 cells, which were then treated with vehicle, TGF-β1 and/or SB431542 as indicated. (**A** and **B**) At the indicated time intervals, Western blotting was performed to analyze the levels of phosphorylated Smad2 and Smad3. GAPDH served as a loading control. **(C)** Cells were co-treated with or without TGF-β1 (200 pM) and SB431542 (5 μM) for 24 h. Western blotting was performed to assess the expression of phosphorylated Smad2 and Smad3 and GAPDH. **(D)** Cells were either untreated or induced with TGF-β1 (200 pM) for 24 h. Total RNA was extracted, and the expression of TGF-β1, CTGF and PAI-1 was assessed by real-time RT-PCR.

To explore the mechanism that leads to increased TGF-β-induced transcriptional activities after the downregulation of PPM1A, we evaluated whether PPM1A could inhibit TGF-β signaling in T24 and 5637 BCa cells. First, the expression levels of PPM1A related TGF-β signaling components, including TβRII, TβRI, Smad2, Smad3 and Smad4, were assessed in these two cell lines (Supplementary Figure 1C and 1D). PPM1A RNAi and control RNAi cells were treated with TGF-β1, and we investigated the temporal pattern of Smad2/3 dephosphorylation. Upon stimulation with TGF-β1, we found that the levels of phosphorylated Smad2/3 were significantly lower in control cells than in PPM1A knockdown cells (Figure 4A, Figure 4B). In addition, we confirmed that the TβRI kinase inhibitor SB431542 could effectively eliminate the increased phosphorylation of Smad2/3 induced by TGF-β1 in PPM1A-knockdown cells (Figure 4C). To test whether the downregulation of PPM1A compromised TGF-β signaling, we measured its effects on the TGF-β stimulation of PAI-1 and CTGF, the target genes of TGF-β that are widely used and highly TGF-β responsive, by using real-time PCR analysis. The induction of PAI-1 and CTGF was very weak and transient in both cell lines. By comparison, cells with PPM1A downregulation showed strong induction of PAI-1 and CTGF messenger RNA after TGF-β1 stimulation (Figure 4D). This result suggests that PPM1A can reduce the level of p-Smad2/3 induced by TGF-β1, and this effect could be blocked by treatment with a TβRI kinase inhibitor.

### PPM1A inhibited BCa invasiveness/metastasis in a TGF-β/Smad-dependent manner

**Figure 5:**
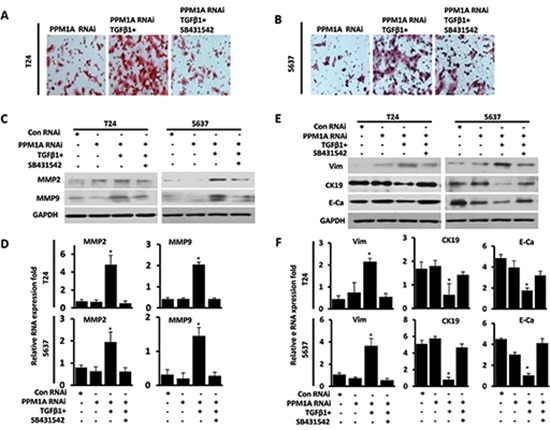
PPM1A inhibited BCa cells invasion and EMT, which was dependent on TGF-β/Smad signaling PPM1A was silenced in T24 and 5637 cells, treated with vehicle, TGF-β1 and/or SB431542, as indicated. (**A** and **B**) The number of cells that had invaded through the membrane with 1% gelatin was counted. **(C)** Western blotting analysis of the expression of MMP2 and MMP9. GAPDH was used as a loading control. **(D)** mRNA was extracted, and MMP2 and MMP9 expression was analyzed by real-time RT-PCR. **(E)** Western blotting analysis of the expression of E-cadherin, CK19 and vimentin. GAPDH was used as a loading control. F. mRNA was extracted, and the expression of vimentin, CK19 and E-cadherin was analyzed by real-time RT-PCR.

Having shown that PPM1A could suppress cell migration and invasion and that PPM1A could block TGF-β signaling by dephosphorylating TGF-β-activated Smad2/3 in BCa cells, we next sought to investigate whether the TGF-β/Smad signaling pathway mediates the suppression of invasion driven by PPM1A in BCa. Migration and invasion assays showed that TGF-β1 treatment promoted cell migration and invasion in PPM1A RNAi BCa cells, whereas co-treatment with the type I receptor inhibitor SB431542 significantly abrogated the pro-migration and invasion effects induced by TGF-β1 in PPM1A knockdown cells (Figure 5A, Figure 5B and Supplementary Figure 2B). To eliminate possible off-target effects of SB431542, SD-208, a 2,4-disubstituted pteridine and ATP-competitive inhibitor of TGF-β receptor I kinase (TβRI), was used to inhibit TGF-β signal pathway activation in parallel. Transwell assays showed that TGF-β1 treatment promoted cell invasion in PPM1A-knockdown BCa cells, and co-treatment with the type I receptor inhibitors SB431542 or SD-208 consistently abrogated the cell invasion activities induced by TGF-β1 in PPM1A-knockdown cells (Supplementary Figure 3). Furthermore, in PPM1A knockdown cells, TGF-β1 treatment led to a significant increase in the expression of MMP2 and MMP9 in both cell lines, which could also be prevented by SB431542, as shown by Western blot and real-time RT-PCR analyses (Figure 5C, Figure 5D). Taken together, our results suggest that PPM1A inhibits migration and invasion and that this effect is dependent on TGF-β/Smad signaling.

### Downregulation of PPM1A significantly promoted TGF-β1-dependent EMT *in vitro*

The EMT is a critical step in TGF-β-induced cancer cell migration and invasion [19]. Phenotypically, EMT in response to TGF-β1 signaling is characterized by the downregulation of epithelial markers and the upregulation of mesenchymal markers [10]. In this study, we observed the morphology of PPM1A RNAi and control T24 and 5637 cells, which were treated with TGF-β1 or vehicle, by fluorescence microscopy. We found that while T24 and 5627 cells grew as tightly packed colonies characteristic of epithelial cells and showed flattened spreading, PPM1A RNAi cells treated with TGF-β1 were spindle-shaped, showed active spreading and had lost the majority of their cell-cell contacts (Supplementary Figure 2A). Consistent with these observations, we found that only in the presence of TGF-β1, PPM1A knockdown led to a significant reduction in the expression of CK19 and E-cadherin and increased the expression of Vimentin in both cell lines. Additionally, these effects were prevented by treatment with the SB431542, as shown by Western blot and real-time RT-PCR analysis (Figure 5E, Figure 5F). These results suggest that upon activation of TGF-β signaling induced by PPM1A, cells exhibit a more overt EMT, which may contribute to the formation of more aggressive and metastatic tumors. These results indicated that the reduced expression of PPM1A promoted the TGF-β1-induced EMT of tumor cells *in vitro*. As such, PPM1A represents a potential key regulatory factor of TGF-β1-induced human BCa invasion and EMT.

### Correlations between PPM1A and biomarkers related to the TGF-β signaling pathway and tumor invasion in BCa samples

**Figure 6:**
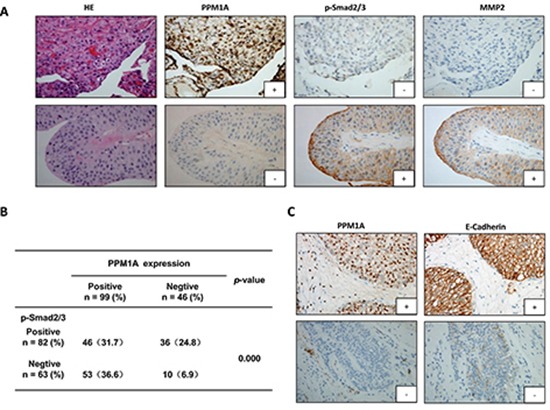
Immunohistochemical staining for PPM1A, p-Smad2/3, MMP2 and E-cadherin in 145 BCa samples **(A)** Representative images of hematoxylin and eosin staining and IHC for PPM1A, p-Smad2/3 and MMP2 from consecutive sections are shown (× 200). **(B)** The correlation between PPM1A and p-Smad2/3 was analyzed in tissues from 145 cases of BCa. **(C)** Representative IHC images for PPM1A and E-cadherin from consecutive sections are shown (× 200).

To verify the *in vitro* and *in vivo* results reported above, we performed immunohistochemical staining for PPM1A and biomarkers related to the TGF-β signaling pathway and cell invasiveness, metastasis and EMT in 145 BCa samples. Representative immunostaining of PPM1A and the associated positive biomarkers is shown in Figure 6A. In the BCa samples, we observed that the loss of PPM1A expression was significantly correlated with high p-Smad2/3 expression (*p* < 0.001), but there was no statistically significant association with Smad2/3 (*p* = 0.311) (Supplementary Table 3). Pearson's correlation analysis showed that decreased PPM1A was significantly correlated with elevated MMP2 (*p* = 0.000), MMP9 (*p* = 0.029) and the loss of PPM1A expression was significantly correlated with low E-cadherin expression (*p* = 0.000) (Figure 6A, Figure 6B and Supplementary Table 3). However, we found that there were no significant correlations between PPM1A and Ki67, CK19 or Vimentin (Supplementary Table 3). Consistent with our previous *in vitro* and *in vivo* results, these correlations between PPM1A and the biomarkers detected in the tissue samples indicated that the deficiency PPM1A expression promoted TGF-β1-induced p-Smad2/3 and stimulated TGF-β signaling pathway-induced migration, invasion and EMT in BCa.

## DISCUSSION

There are two subtypes of bladder urothelial carcinomas, superficial and muscle-invasive cancers, which are distinguished according to whether the tumor infiltration extends to the muscular bladder wall [20]. BCa carries a high risk of recurrence and poor prognosis due to muscle invasion and metastasis [21]. TGF-β is believed to contribute to carcinoma development through the promotion of metastasis and induction of the EMT [7]. We previously reported that Ski, as a negative regulator of TGF-β signaling, suppresses cell invasion and metastasis [22]. Cumulative investigations showed that blockade of TGF-β signaling using inhibitors significantly suppresses tumor invasiveness and metastasis [23-26]. In BCa, it has been demonstrated that the serum level and tissue expression of TGF-β1 is significantly elevated [27, 28], and increased levels of TGF-β in patients with bladder cancer have significant prognostic value for highly aggressive metastatic disease and are considered a poor prognosis marker [8, 29].

The activity of the TGF-β-initiated signaling pathway is under tight control by the level of activated R-Smads [30, 31]. PPM1A has recently been reported to act as an antagonist of TGF-β signaling by dephosphorylating TGF-β-activated Smad2/3 [15]. PPM1A belongs to the PP2C phosphatase family and specifically interacts with and dephosphorylates phosphorylated substrates. Several kinases, including p38 and MAPK [32], CDK9 [33], PI3K [34], and NF-kB [35], have been identified to be substrates of PPM1A. However, the distinct functions of PPM1A activity and how PPM1A regulates tumor cell activity remain largely unknown. This study describes the functions of PPM1A in the progression of BCa and highlights the potential roles of PPM1A in BCa cell migration, invasion and EMT induced by TGF-β1. We evaluated the expression status of PPM1A in BCa tissue samples and found that PPM1A is a prognostic factor that is especially associated with the development of tumor invasion in bladder cancer patients.

PPM1A has a large number of substrates and exhibits distinct functions in different cell types and contexts. However, how PPM1A regulated during tumor invasion remains to be elucidated. In our study, we found that knockdown of PPM1A has little effect on cell migration and invasiveness, but when PPM1A-knockdown BCa cells are administered TGF-β1 treatment, cell migration and invasiveness are significantly enhanced compared to control cells. Based on these investigations, we conclude that knockdown of endogenous PPM1A sensitizes cell migration and invasion to respond to TGF-β1. We further established a subcutaneous xenotransplantation BCa model and lung metastasis model. Consistent with our *in vitro* results, significant reductions in tumor growth and metastasis were observed following PPM1A overexpression.

Smads possess intrinsic nucleocytoplasmic shuttling capacity, which enables them to transmit TGF-β signals from the cell membrane to the nucleus; when these activated pathways are terminated, the majority of activated R-Smads are not degraded, but recycled [36]. Dai *et al.* recently discovered that the first step in the termination of nuclear Smad signaling is initiated by PPM1A, which dephosphorylates Smad2/3 in the nucleus [37], and that dephosphorylation is a prerequisite for the recycling of R-Smads [38]. PPM1A has been demonstrated to physically interact with p-Smad2 and p-Smad3 [15]. Here, we present studies on PPM1A-mediated dephosphorylation of p-Smad2/3 in BCa cell lines. We found that upon stimulation with TGF-β1, the levels of phosphorylated Smad2/3 are significantly higher and sustained in PPM1A knockdown cells than in control cells. We further found that cells downregulated for PPM1A show strong induction of PAI-1 and CTGF mRNA after TGF-β1 stimulation. In the BCa samples, we observed that the loss of PPM1A expression was significantly correlated with high p-Smad2/3 expression. All of these results lead to the conclusion that PPM1A inhibits the TGF-β-induced the activity of Smad2, Smad3 and transcriotional responses, whereas depletion of PPM1A enhances the activation of TGF-β/Smads signaling in BCa cells.

Previous studies have shown that lower expression of PPM1A is involved in human cytotrophoblast cell invasion and migration [16, 39] and that decreased PPM1A expression inhibits prostate cancer metastases [35]. Our studies demonstrate that the TGF-β signaling pathway is essential for PPM1A to prevent invasion and metastasis of BCa. In addition to TGF-β1 treatment resulting in a significant effect on migration and invasiveness *in vitro*, we found that co-treatment withthe inhibitors of TβRI (SB431542) significantly abrogated the pro-migration and invasion effects of PPM1A downregulation.

One mechanism by which TGF-β contributes to cancer progression is through induction of the EMT, which is a critical step in TGF-β-induced cancer cell migration and invasion [40, 41]. This process involves the disaggregation of structured epithelial units to enable cell motility and morphogenesis during embryonic development and has also attracted attention in studies of tumor progression [42, 43] [44]. One of our most notable observations was that PPM1A RNAi cells treated with TGF-β1 showed a mesenchymal phenotype. Furthermore, with TGF-β1 stimulation, PPM1A knockdown led to a significant reduction in the expression of epithelial markers and increased expression of mesenchymal markers in both cell lines, and these effects were prevented by treatment with SB431542, suggesting that upon activation of TGF-β signaling induced by loss of PPM1A, cells exhibit a more overt EMT, which may contribute to the formation of more aggressive and metastatic tumors.

The matrix metalloproteinase (MMP) family is known to degrade nearly all components of the ECM. MMP-2 and MMP-9, the most extensively studied MMPs in tumor invasion, mainly degrade collagen IV and a number of other ECM proteins [45, 46]. We found that in PPM1A-knockdown BCa cells, TGF-β1 treatment induced a significant increase in the expression of MMP2 and MMP9, which could also be prevented by SB431542. In our study, we further found significant correlations between PPM1A expression and MMP2, MMP9 and E-Cadherin in BCa samples. These findings are consistent with our previous observations from *in vitro* and *in vivo* studies.

In the present study, we revealed a previously unrecognized role for the protein phosphatase PPM1A in BCa, serving as a modifier of TGF-β signaling through control of the phosphorylation of Smad2/3. Additionally, PPM1A was shown to suppress BCa cell migration, invasion and EMT development and this effect was dependent on TGF-β/Smad signaling. Furthermore, loss of PPM1A expression correlated with poor differentiation, the development of muscle-invasive tumors and poor prognosis in patients with BCa. Therefore, our findings suggest a notable tumor-promoting role for PPM1A that is dependent on the TGF-β/Smad signaling pathway in BCa.

## MATERIALS AND METHODS

### Patients and clinical specimens

Formalin-fixed, paraffin-embedded tissues were collected from 145 BCa patients. These patients were enrolled from January 2005 to December 2008 at the Department of Urology at the Tenth People's Hospital, which is affiliated with the Tongji University of Medicine (41 tumors), and the Department of Pathology of Huashan Hospital, which is affiliated with Fudan University (104 tumors) (ShangHai, China). All tissue samples were cut into 3-μm-thick sections, which were subsequently stained with hematoxylin and eosin. Two experienced pathologists confirmed the diagnosis of “urothelial carcinoma of the bladder” for all specimens. None of the patients had received neoadjuvant radio/chemotherapy.

### Cell lines and mice

The human BCa cell lines SW780, UM-UC-3, J82, 5637 and T24 were used in this study. The cell lines SW780, UM-UC-3, and J82 were obtained from the cell bank of the Chinese Academy of Sciences, and 5637 and T24 cells were obtained from the American Type Culture Collection. The cells were grown in complete growth medium, as recommended by the manufacturer. Cultured cells were maintained in a humidified, 5% CO_2_ atmosphere at 37 °C. All of the cell lines were regularly authenticated by checking their morphology and were tested for the absence of mycoplasma contamination (MycoAlert, Lonza, Rockland, ME, USA).

Male BALB/c-nu mice (4–5 weeks of age, 18–20 g) were obtained from the Shanghai SLAC Laboratory Animal Co. Ltd. (Shanghai, China) and housed in laminar flow cabinets under specific pathogen-free conditions with food and water provided *ad libitum*. All of the mouse experiments were conducted in accordance with the NIH guidelines for the Care and Use of Laboratory Animals. The study protocol was also approved by the Committee on the Use of Live Animals in Teaching and Research, Fudan University, Shanghai.

### Reagents

Human recombinant TGF-β1 was obtained from R&D Systems (Minneapolis, MN). The TGF-β RI (TβRI) kinase inhibitor SB431542 was obtained from Millipore (Billerica, MA), and SD-208 was purchased from Sigma (St Louis, MO). The following antibodies were used in this study: anti-PPM1A, anti-MMP2, anti-MMP9, anti-Smad2, anti-Smad3, anti-phosphorylated Smad2 and anti-phosphorylated Smad3 (Cell Signaling Technology, Beverly, MA) as well as anti-E-cadherin, anti-GAPDH, anti-CK19, anti-phosphorylated Smad2/3 and anti-Vim (Santa Cruz, CA, USA). SB431542 was applied during the TGF-β1 perfusion.

### Lentivirus-mediated PPM1A RNA interference

We established stable, PPM1A-silenced T24 and 5637 transfectants by infecting cells with lentivirus encoding shPPM1A (purchased from GenePharma, Shanghai, China). The lentivirus packaging shRNA expression vector was established as previously reported [47]. Briefly, the sequences for targeting the PPM1A gene (GenBank accession no. NM_021003) were selected using the BLOCK-iT RNAi Designer (Invitrogen, Carlsbad, CA). Then, three short hairpin RNA oligonucleotides were designed (RNAi #1: 5'-GTCGACACCTGTTTGTATA-3'; RNAi #2: 5'-CTGGGATGTTATGGGAAAT-3'; RNAi #3: 5'-GCTGTGAGCATTTGTTAGA-3') and cloned into the pGLV-U6-GFP lentivirus vector (GenePharma, Shanghai, China). The negative control vector contained a nonsense shRNA (5'- TTCTCCGAACGTGTCACGT-3') to control for any non-RNAi-mediated effects. T24 and 5637 cells were exposed to lentivirus-containing supernatant for 24 h in the presence of polybrene (Sigma). Stable transfectants were selected with puromycin (2 mg/ml) and verified by Western blotting and real-time PCR analysis.

### Cell transfections

Wild-type human PPM1A (Hu-PPM1A) cDNA was cloned into the pcDNA-DEST40 vector (Invitrogen, San Diego, CA) according to the manufacturer's recommendations. All transfections were carried out using the Lipofectamine 2000 Transfection Reagent (Invitrogen, Carlsbad, USA).

### Wound closure assays

The cells were plated in the wells of 6-well plates. Confluent cell monolayers were wounded by manually drawing a furrow across the monolayer with a 10-μl pipette tip. The cell culture medium was then replaced with fresh medium, and 200 pM TGF-β1 or vehicle was added as required. Wound closure was then monitored at various time points by phase contrast microscopy. The wound area at each time point after wounding was quantified using Adobe Photoshop version 7.0 (Adobe Systems Inc.) and Image version 1.29 (NIH) software. The experiments were performed in duplicate.

### Invasion assay

Cell invasion was assessed using the transwell chamber invasion assay (Matrigel-coated membrane, BD Biosciences) with 24-well transwell units with polycarbonate filters (pore size 8 mm) coated on the upper side with 1% gelatin (Sigma). Cells (1 × 10^5^) were seeded in serum-free medium into the upper chamber and allowed to invade into the lower chamber, which contained 10% FCS as a chemoattractant. TGF-β1 (200 pM) or vehicle alone was added to the upper and lower chambers. After 48 h, cells that had invaded through the Matrigel matrix and adhered to the underside of the membrane were counted.

### Xenograft studies

Briefly, mice were randomly divided into four groups consisting of six mice each. Cells (5x10^6^ cells in 200 μl) were suspended in RPMI 1640 medium and injected subcutaneously into the flank of each BALB/c nude mouse. The length and width of the resulting tumors (in millimeters) were measured every three days with calipers. The tumor diameter was measured, and the volume (length × width^2^ × 0.52) was calculated. The mice were sacrificed humanely on day 40, and the tumors were dissected and weighed. Then, the tumors were fixed, embedded and cut into 3-μm-thick sections, which were subsequently stained with hematoxylin and eosin to permit observation of the tumor margin. Immunohistochemistry was also performed on these sections.

### *In vivo* metastasis assay

Male nude mice received intravenous injections of 2 × 10^6^ cells in 0.2 ml of normal saline via the tail vein. Seven weeks after injection, the mice were examined grossly at necropsy for the presence of metastases in the lungs. We evaluated tumor metastasis by counting the number of metastatic colonies in one histologic section from the midportion of each lung sample from each mouse. In particular, we calculated the ratio of the metastatic area to the total area in histologic sections from the midportion of each lung [48]. The ratio of the metastatic area to the total area in a histologic section was calculated using Adobe Photoshop version 7.0 (Adobe Systems Inc.) and Image version 1.29 (NIH) software.

### Real-time RT-PCR analysis

Briefly, total RNA was isolated from cultured cells or tissues using the TRIzol^®^ Reagent (Invitrogen, San Diego, CA, USA) according to the manufacturer's instructions. Real-time RT-PCR using SYBR green I was carried out to compare the relative expression of specific mRNAs, as previously described. The primer sequences are described in Supplementary Table 4.

### Western blot analysis

Proteins were extracted from cultured cells and then quantitated using the bicinchoninic acid (BCa) assay kit (Pierce, Rockford, IL, USA) with BSA as a standard. Equal amounts of protein from different samples were separated by 10% SDS-PAGE and then incubated with antihuman monoclonal antibodies. Target proteins were detected using the enhanced chemiluminescence (ECL) kit (Amersham Pharmacia Biotech, Uppsala, Sweden) with exposure to X-ray films (Eastman Kodak, Rochester, NY, USA).

### Immunohistochemistry

Specimens of tumor tissue were fixed in 10% formalin and embedded in paraffin wax. Three-micrometer sections were then cut from the paraffin blocks for immunohistochemical (IHC) analysis. The sections were stained with anti-PPM1A (1:200), anti-E-Cadherin (1:100), anti-CK19 (1:500), anti-Vimentin (1:100), anti-MMP2 (1:100) and anti-MMP9 (1:100) antibodies at 4°C overnight. The secondary antibody and avidin-biotin peroxidase complex method was used according to the standard protocols provided by the manufacturer (Vector Laboratories, CA). An immunoglobulin-negative control was used to rule out non-specific binding. These procedures were performed by two independent investigators and one pathologist who were blinded to the model/treatment type for the series of specimens.

### Statistical analysis

ANOVA and the Student's t test were used to determine statistically significant differences between experimental groups. The Kaplan-Meier method was used to calculate the overall survival rate, and the prognostic significance was evaluated by the log-rank test. The correlation of PPM1A immunoreactivity with the patients' clinicopathological variables was analyzed using Fisher's exact test. Differences were considered significant at *p* < 0.05.

## SUPPLEMENTARY FIGURES AND TABLES


