# Supplementary material for: Loss of PPM1A expression enhances invasion and the epithelial-to-mesenchymal transition in bladder cancer by activating the TGF-β/Smad signaling pathway
Source: Oncotarget. 2014 Jul 1;5(14):5700–11. doi: 10.18632/oncotarget.2144 (PMC4170610; doi:10.18632/oncotarget.2144)
Supplement: Supplementary file 1 [file oncotarget-05-5700-s001.pdf]

SUPPLEMENTARY FIGURES AND TABLES

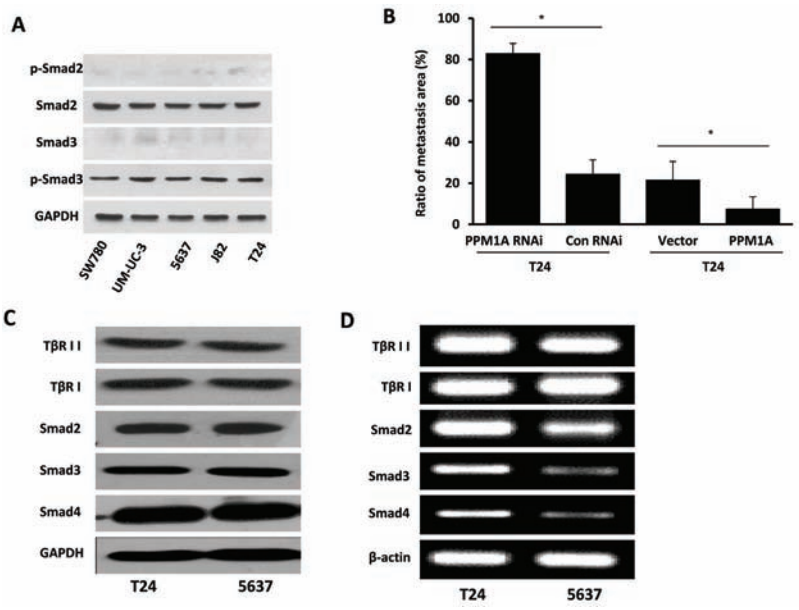

Supplementary Figure 1: Expression of PPM1A and TGF-β pathway components in human bladder cancer cell lines. **(A)** Western blotting was performed to analyze the levels of activation of Smad2 and Smad3 in five bladder cancer cell lines. **(B)** Western blot showing the expression of TGF-β pathway components in T24 and 5637. **(C)** RT-PCR analysis showing the expression of TGF-β pathway components in T24 and 5637. **(D)** The metastatic lesions were quantitatively evaluated by the number of metastatic area to total area in lungs of mice. Compare the lung metastatic nodules of T24 vector, T24 PPM1A, T24 RNAi control and T24 PPM1A RNAi cells into the tail veins of nude mice. Mice injected with PPM1A-knockdown cells developed significantly more lung metastases than mice injected with T24 PPM1A and control cells.

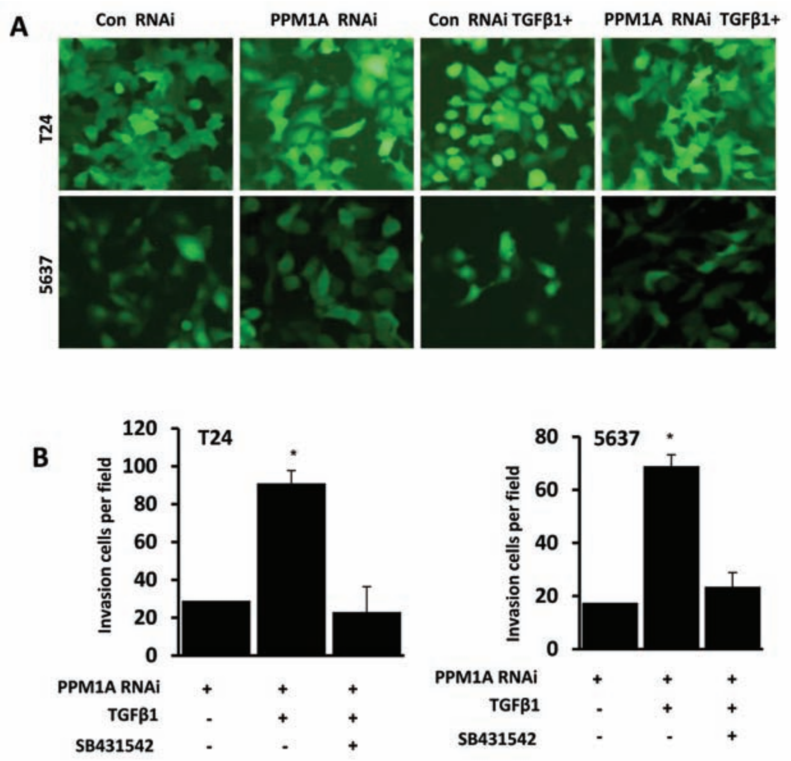

Supplementary Figure 2: PPM1A suppresses T24 cell migration in vitro and in vivo. (A) Morphologic change was examined by fluorescence photomicrographs. **(B)** Cell invasive rates of bladder cancer cells treated with TGF-β1 (200pM) or SB431542 were compared via Matrigel invasion assays in T24 and 5637 cells assays.

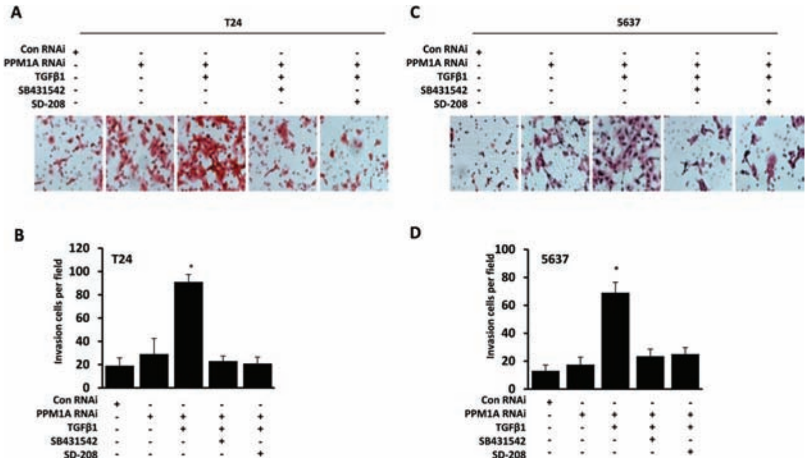

Supplementary Figure 3: PPM1A inhibited BCa cells invasion which was dependent on TGF-β/Smad signaling. PPM1A was silenced in T24 and 5637 cells, treated with vehicle, TGF-β1 and/or SB431542, SD-208(1μM), as indicated. **(A** and **B)** The number of T24 that had invaded through the transwell membrane with 1% gelatin was counted. **(C** and **D)** The number of 5637 that had invaded through the transwell membrane with 1% gelatin was counted.

Supplementary Table S1. Clinicopathologic characteristics of patients with PPM1A expression

Factors	Number	PPM1A positive	PPM1A negtive	p
**Age at diagnosis, mean±SD**	145	64.4±13.0 n=99	67.2±11.4 n=46	0.203
**Gender**				
Male	125	84	41	0.609
Female	20	15	5	
**Histological grade**				
Low	85	51	34	0.012
High	60	48	12	
**T stage**				
Ta Tis/T1	95	59	36	0.038
T2/T3	50	40	10	
**N status**				
N0	112	79	33	0.294
N1,N2	33	20	13	
**Tumor recurrence**				
Yes	36	25	11	0.518
No	109	74	35	

Supplementary Table S2. Clinicopathologic characteristics of patients with PPM1A expression

patient	Age (years)	sex	primary tumor	recurrence tumor	time of recurrence (month)	the expression of PPM1A in primary tumors	the expression of PPM1A in recurrence tumors	*p* value
1	56	F	1	2	6	P	N	
2	67	M	1	2	8	P	N	
3	46	M	1	2	9	N	N	
4	76	M	1	2	12	P	N	
5	79	F	1	2	23	P	N	0.012
6	66	F	1	2	18	P	N	
7	53	F	1	2	17	P	P	
8	58	M	1	2	15	P	P	
9	79	M	1	2	23	P	N	
10	80	F	1	2	7	N	N	
1	47	F	1	1	7	N	P	
2	67	F	1	1	9	P	P	
3	59	F	1	1	6	P	P	
4	69	M	1	1	11	N	N	
5	63	M	1	1	23	N	P	0.314
6	78	F	1	1	18	P	P	
7	79	F	1	1	16	N	N	
8	71	M	1	1	12	P	P	
9	81	M	1	1	17	P	P	
10	74	M	1	1	19	P	P	

F = Female, M = male, 1 = Superficial Bca, 2 = Muscle-invasive Bca, P = Positive, N = Negative

Supplementary Table S3. commonly used biomarkers with PPM1A expression

Factors	Number	PPM1A positive	PPM1A negtive	p
**MMP2**				
positive	48	17	31	0.000
negtive	97	82	15	
**MMP9**				
positive	59	34	25	0.029
negtive	86	65	21	
**Ki67**				
≥10%+	94	65	29	0.450
<10%+	51	34	17	
**E-cadherin**				
positive	72	62	10	0.000
negtive	73	37	36	
**Vimentin**				
positive	41	30	11	0.278
negtive	104	69	35	
**CK19**				
positive	129	90	39	0.271
negtive	16	9	7	

Supplementary Table S4. Primers for RT–PCR and their lengths of product

Gene	Primer sequence		Product size (bp)
**TβRI**	F	5’- TGTGAAGCCTTGAGAGTAATG -3’	113
	R	5’- TGTTGACTGAGTTGCGATAA -3’	
**TβRII**	F	5’- CAACAACATCAACCACAAC -A3’	84
	R	5’- TTATAGACCTCAGCAAAGCGA -3’	
**CTGF**	F	5’- ACAGTCCGTCAAAACAGATT -3’	92
	R	5’- AAAAGTGAGGCTACCACATT -3’	
**CDK2**	F	5’-CCT GGA GAT TCT GAG ATT GAC-3’	114
	R	5’- GGG GAA ACT TGG CTT GTA A -3’	
**PAI-1**	F	5’- CCCTTGAGTGCTTGTTAGAGA -3’	112
	R	5’- GGCTGGACTTCCTGAGATAC -3’	
**PPM1A**	F	5’- CGCTGGAGAAAGAACGAAT -3’	158
	R	5’- GACTTCAGGCTCTGGTGAGAC -3’	
**Smad2**	F	5’- GTGAAGATGGAGAAACAAGTG -3’	91
	R	5’- ACAGGGGAAAGAGTAGTAGGA -3’	
**Smad3**	F	5’- GGCTGCTCTCCAATGTCAAC -3’	94
	R	5’- ACCTCCCCTCCGATGTAGTAG -3’	
**Smad4**	F	5’- GGACTTCAGGGGCTTCTAA -3’	167
	R	5’- AAGGAGGCACACTAACACTCA -3’	
**VEGF**	F	5’- CAGCAGAAAGAGGAAAGAGGT -3’	130
	R	5’- CCAAAAGCAGGTCACTCACT -3’	
**E-Cadherin**	F	5’- AAGACAAAGAAGGCAAGGTT -3’	148
	R	5’- AAGAGAGTGTATGTGGCAATG -3’	
**MMP2**	F	5’- GTGCTGAAGGACACACTAAAG-3’	143
	R	5’- TTGCGAGGGAAGAAGTTG -3’	
**MMP9**	F	5’-CCAAAACTACTCGGAAGACTTGC-3’	208
	R	5’- AGGAGGAAAGGCGTGTGC -3’	
**CK19**	F	5’- GCGGGACAAGATTCTTGGTG -3’	214
	R	5’- CTTCAGGCCTTCGATCTGCAT-3’	
**Vimentin**	F	5’- CTCTTCTCCGGGAGCCAGTC -3’	188
	R	5’- CTGCCCAGGCTGTAGGTG -3’	
**β-actin**	F	5’- TGACGTGGACATCCGCAAAG -3’	150
	R	5’- CTGGAAGGTGGACAGCGAGG -3’	
